# Bayesian Correlation Analysis for Sequence Count Data

**DOI:** 10.1371/journal.pone.0163595

**Published:** 2016-10-04

**Authors:** Daniel Sánchez-Taltavull, Parameswaran Ramachandran, Nelson Lau, Theodore J. Perkins

**Affiliations:** 1 Regenerative Medicine Program, Ottawa Hospital Research Institute, Ottawa, Ontario, Canada; 2 Department of Biochemistry, Microbiology and Immunology, University of Ottawa, Ottawa, Ontario, Canada; Hospital for Sick Children, CANADA

## Abstract

Evaluating the similarity of different measured variables is a fundamental task of statistics, and a key part of many bioinformatics algorithms. Here we propose a Bayesian scheme for estimating the correlation between different entities’ measurements based on high-throughput sequencing data. These entities could be different genes or miRNAs whose expression is measured by RNA-seq, different transcription factors or histone marks whose expression is measured by ChIP-seq, or even combinations of different types of entities. Our Bayesian formulation accounts for both measured signal levels and uncertainty in those levels, due to varying sequencing depth in different experiments and to varying absolute levels of individual entities, both of which affect the precision of the measurements. In comparison with a traditional Pearson correlation analysis, we show that our Bayesian correlation analysis retains high correlations when measurement confidence is high, but suppresses correlations when measurement confidence is low—especially for entities with low signal levels. In addition, we consider the influence of priors on the Bayesian correlation estimate. Perhaps surprisingly, we show that naive, uniform priors on entities’ signal levels can lead to highly biased correlation estimates, particularly when different experiments have widely varying sequencing depths. However, we propose two alternative priors that provably mitigate this problem. We also prove that, like traditional Pearson correlation, our Bayesian correlation calculation constitutes a kernel in the machine learning sense, and thus can be used as a similarity measure in any kernel-based machine learning algorithm. We demonstrate our approach on two RNA-seq datasets and one miRNA-seq dataset.

## Introduction

A fundamental task in data analysis is to assess the relatedness of different measured variables. In bioinformatics, for example, we may want to know which genes have similar patterns of expression across conditions [1, 2], which biomarkers correlate to cancer patient survival [3, 4], or which patterns of histone modification reflect to cell identity [5]. Standard linear correlation analysis is often used to assess relatedness, or is used as a basis for more sophisticated analysis, such as the construction of Relevance Networks [6]. Indeed, some notion of similarity underlies many machine learning algorithms, including clustering, principal components analysis, regression, etc. Kernel-based methods make the central role of similarity most explicit [7, 8], but it is important in essentially all algorithms. In this work, we propose a general approach for assessing similarity of variables measured by high-throughput sequencing.

High-throughput sequencing (HTS), of course, has become a central technology driving molecular biology research. For example, it was the basis for the high-profile ENCODE and modENCODE projects aimed at understanding gene regulatory networks on a large scale [9–11]. Similarly, it has been used to generate numerous insights into cancer biology and biomarkers related to survival [12, 13]. The flexibility of the technology allows it to be used for measuring many different things: gene and microRNA expression, transcription factor binding and histone modifications, DNA methylation, and so on [14]. The costs of HTS experiments are generally still higher than for the microarray-based technologies they are starting to displace. However, HTS is often favored because of its greater dynamic range [15, 16] and because it can truly measure across the whole genome, whereas array-based methods—by virtue of choosing probes—must preselect what portions of the genome or transcriptome are measured.

It is important to realize, however, that the precision of sequencing-based measurements varies for different experiments and for different things being measured in those experiments. To understand this, consider that in many cases the outcome of a set of sequencing experiments can be summarized in a matrix of count data. The rows of the matrix correspond to different biological entities being measured. For instance, in an RNA-seq dataset, each row may correspond to a different gene whose expression is being measured, or perhaps different transcripts. In a miRNA-seq dataset, each row corresponds to a different microRNA. In a ChIP-seq dataset, each row may represent a different region of the genome. The columns of the matrix typically correspond to different conditions or factors being measured. For example, they might represent different tissues or cell types in which expression is being measured, different drug treatments, or different factors that are being assayed by ChIP-seq. Some or all of the columns might be replicate measurements of the same condition. In this paper we will use *R* to denote the read count matrix, with *R*_*ic*_ denoting the counts attributed to entity *i* under condition *c*, and *R*_*c*_ denoting the total counts for condition *c* (that is, the column sum). How counts are attributed to entities depends on the nature of the data, and is not something we address. Often the counts are whole numbers, 0, 1, 2, …, although sometimes fractional counts are used when attribution is uncertain. We will require only that the counts are non-negative, and we will use the term “count” even if some matrix entries are fractional.

**Fig 1:**
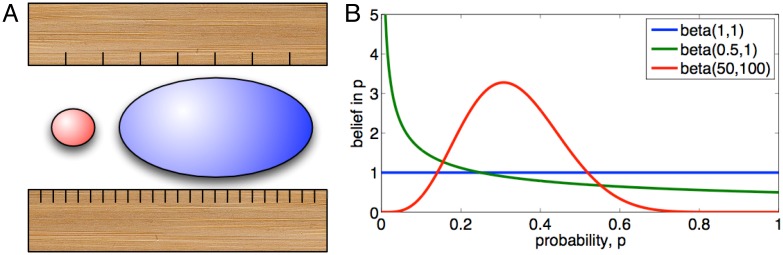
Concepts in the precision of high-throughput sequencing count data. (A) Deeper sequencing is analogous to measuring objects using a ruler wither finer gradations—all objects are measured with greater absolute precision. However, regardless of sequencing depth, lower count entities (e.g. low expression genes or miRNAs) are measured with less relative precision than higher count entities, similar to the red and blue objects respectively. (B) The Beta distribution can represent our belief over the true, unknown expression levels of gene or miRNAs as a fraction of total expression (for example).

The precision of any scientific experiment varies due to many factors, but even at the very abstract level of the count matrix *R*, we can identify two important influences on precision. One is that the total reads sequenced and attributed, *R*_*c*_, can vary by condition *c*. The greater *R*_*c*_ is, that is, the deeper the sequencing, the greater the precision with which every entity is being measured under that condition. To make an analogy, if we think of measuring a physical object with a ruler, and we can choose between a ruler with fewer marks on it and one with more marks—more, finer gradations—then we can make more precise measurements using the ruler with more gradations (Fig 1A).

Another infuence involves the asbolute level (e.g., expression) of the entity being measured. For instance, suppose an entity *i* is attributed just one count out of a million under condition *c*, thus *R*_*ic*_ = 1 and *R*_*c*_ = 10^6^. Intuitively, if we replicated that experiment, we might get one count again, or we might get zero, or two or three, maybe more. Thus, there is substantial uncertainty in the true level of entity *i*—in fact, approximately a two orders of magnitude uncertainty. (One way of formalizing this is to assume that the expected value of *R*_*ic*_ is some true level *T*, and the observed value is binomially distributed with success probability *T*/*R*_*c*_ on *R*_*c*_ tries. For any given value of *T*, we can compute the probability of our observation *R*_*ic*_ = 1, and we can compute the probability of all other possible observed counts, {0, 2, 3, 4, …, *R*_*c*_}. A *p*-value for rejecting a hypothesized value of *T* can be computed by summing only those probabilities that are less than or equal to the probability that *R*_*ic*_ = 1; these are the possible outcomes that are considered “more extreme” than the actual outcome. Performing this calculation for various values of *T*, we found that *T* ∈ [0.0520, 5.7550] would *not* be rejected at the standard *p*-value threshold of 0.05, indicating more than 2 orders of magnitude uncertainty in the true level of entity *i*). On the other hand, imagine *i* is a high-count entity, say receiving *R*_*ic*_ = 10^5^ reads out of *R*_*c*_ = 10^6^. If we were to replicate the experiment, we would not expect to see as few as 10^4^ reads or as many as 10^6^. In fact, we might expect only about a percentage point varability in the measured level. (Repeating the same analysis described above, we find values of *T* in the range [99413, 100589] would not be rejected at *p* = 0.05, but values farther from 10^5^ would be rejected, so that there is only about 1.18% uncertainty in the true level). In essence, because the marks on the ruler—individual reads—are of a fixed size regardless of the size of the object being measured, large objects are measured with much greater relative precision. Or, to put it yet another way, the signal-to-noise ratio is much better for high-count entities than for low-count entities.

How do these considerations on precision influence our ability to estimate similarity between different entities across conditions? Let us consider a simple example. Imagine that we have measured gene expression across three different conditions, each with exactly 10^6^ sequencing depth, so that normalization is not an issue. (Expression data is often converted to reads-per-million, rpm, or fragments per-kilobase-per-million, fpkm, so that changes in sequencing depth alone do not make expression values appear to go up or down). Suppose that a gene *x* has counts (1000, 1000, 10000) across the three conditions, gene *y* has counts (100, 100, 200) and gene *z* has counts (0, 0, 1). A naive Pearson correlation analysis would show that all three genes are perfectly (*r* = 1) correlated with each other. Yet, we know that *z*’s measured counts are more likely to be non-representative than those of *x* or *y*. Its count of 1 under the third condition could be a fluke, as could its zero counts in the first two conditions. Thus, we should have more confidence in the *xy* correlation than the *xz* correlation or the *yz* correlation. More subtly, we should have more confidence in the *xz* correlation than the *yz* correlation because of our greater relative precision in measuring *x* than *y*—even though we shouldn’t have high confidence in either of these correlations. To solve the “problem” of apparent correlations with gene *z*, we could adopt some heuristic rule of discarding genes with low counts. But, exactly what consititues a low count? Is it the average count across conditions, or the total read counts, or the maximum count, or the difference between maximum and minimum counts? And what is the threshold for discarding or keeping a gene? Whatever threshold is chosen, there will always be some genes that only just beat the threshold, and yet those will be treated identically to genes where measurements are of high precision. What is needed is a more principled and more graded way of discounting evidence based on the precision of the individual measurements.

Similar phenomena can occur when sequencing depth is different under different conditions. For instance, suppose the sequencing depths in our three conditions are 10^6^, 10^7^ and 10^6^. Further, suppose two genes, *w* and *x*, have observed counts (10, 0, 0), while two other genes, *y* and *z*, have observed counts (0, 100, 0). The Pearson correlation of *w* and *x* is *r* = 1, as is the correlation of *y* and *z*. This is true with or without normalization to library size, and in fact, all four gene are expressed at the same level, relative to library size. Yet, intuitively, we ought to be more confident in the *yz* correlation than in the *wx* correlation, because the higher sequencing depth in condition two means its measurements have greater precision. In the analysis of gene expression in particular, there is a history of noise modeling both for individual conditions and across replicates [17–19], which then impacts the assessment of differential expression. However, uncertainty affects all types of HTS measurements, not just gene expression, and to our knowledge it has not previously been taken into account rigorously as part of correlation analyses.

In this paper, we lay out a Bayesian scheme for assessing the similarity of different entities measured by HTS across different conditions. In particular, we use Bayesian models to expression our beliefs, including uncertainty, about the true levels of different entities across different conditions, and we show how to use those Bayesian beliefs to estimate the correlations between different entitites. Interestingly, we show that naive, uniform priors for the entities’ levels can lead to highly biased estimates of the correlations between them, specifically when different experiments are sequenced to different depths. However, we show that alternative choices of priors can virtually eliminate this effect. We find that our Bayesian correlation estimate naturally suppresses spurious correlations between entities whose true levels are highly uncertain, without the need for arbitrary thresholding. We also show that our Bayesian correlation estimate constitute a kernel in the machine learning sense, opening the door to its use in a wide variety of kernel-based machine learning algorithms. R code implementing our approach is available as supplementary information (S1 Code) or on the web at http://www.perkinslab.ca/Software.html.

## Results

### Bayesian estimation of true signal levels and their correlations

As described in the Introduction, suppose we have a matrix of counts *R*_*ic*_ for entities *i* ∈ {1, 2, …, *m*} and conditions *c* ∈ {1, …, *k*}, and suppose we want to compute the correlation, or similarity, between two entities *i* and *j* (e.g. two genes). As already mentioned, it does not make sense to correlate the read counts themselves, as mere differences in total read counts *R*_*c*_ per condition can induce read count correlations. Such spurious correlations can be suppressed by read count normalization, with the most straightforward choice being to compute the empirical read fractions $p_{ic}^{emp}=R_{ic}/R_{c}$. We *could* then adopt as our similarity measure the Pearson correlation of $p_{ic}^{emp}$ and $p_{jc}^{emp}$ across conditions *c*. However, we have presented arguments against this approach in the Introduction, as it does not take into acount the precision the measurements—that is, the precision of the $p_{ic}^{emp}$.

Instead, let us pursue a Bayesian approach to this estimation problem. Bayesian analysis of digitial expression data goes back at least to the work of Audic and Claverie [20], and we employ a similar scheme here. First, we consider the precision of our read counts. Although the *R*_*ic*_ are observed data, let us view them as random variables. Specifically, let us assume that any given read in experiment *c* has probability *p*_*ic*_ of being attributed to entity *i*, independent of any other factors. Then, the total number of reads attributed to entity *i* under condition *c* should follow a Binomial distribution with success probability *p*_*ic*_ on *R*_*c*_ tries:

$$R_{ic}∼Bin\left(R_{c},p_{ic}\right)=\left(\begin{array}{c} R_{c}\\ R_{ic} \end{array}\right)p_{ic}^{R_{ic}}{\left(1-p_{ic}\right)}^{R_{c}-R_{ic}},$$ (1)

Although *p*_*ic*_ is unknown, we can use the observed data to estimate it. In particular, the standard Bayesian approach would be to adopt prior beliefs over the unknown *p*_*ic*_—that is, to define the likelihoods of different true read fractions before the data is observed—and then compute posterior beliefs based on the data. This could be done through a high-dimensional Dirichlet belief distribution. However, for the sake of simplicity, we will assume independent Beta distributions for each entity and each condition. The Beta distribution is defined by two parameters, *α* and *β*. These two parameters encode both our “estimate” of an unknown success probability *p* and our uncertainty in that estimate. (See also Fig 1B). Intuitively, we tend to believe *p* is large when *α* is large relative to *β*, and we tend to believe that *p* is small when *β* is large relative to *α*. We are uncertain in these beliefs when the total *α* + *β* is small, and we are more certain when *α* + *β* is large. More formally, letting Γ(⋅) denote the gamma function, the belief over *p* given belief parameters *α*_*ic*_ and *β*_*ic*_ is given by:

$$p∼Beta\left(\alpha ,\beta \right)=\frac{\Gamma (\alpha +\beta )}{\Gamma (\alpha )\Gamma (\beta )}p^{\alpha -1}{\left(1-p\right)}^{\beta -1}$$ (2)

In Bayesian approaches to estimation, we begin with a prior belief that holds before we account for the data, and then we use the data to update our belief, resulting in a posterior belief. Having made the Beta assumption, our prior belief for *p*_*ic*_ is specified by entity- and condition-specific prior belief parameters $\alpha_{ic}^{0}$ and $\beta_{ic}^{0}$, while, conveniently, our posterior belief is specified by posterior parameters $\alpha_{ic}=\alpha_{ic}^{0}+R_{ic}$ and $\beta_{ic}=\beta_{ic}^{0}+R_{c}-R_{ic}$. The mean of that posterior belief can be taken as our Bayesian estimate of the true read fraction, and can be written in terms of the read counts and prior parameters. Because we will shortly be treating the condition *c* as if it too is a random variable, let us be clear and write the posterior mean for entity *i*’s true read fraction under condition *c* as

$$E\left(p_{ic}|c\right)=\frac{\alpha_{ic}}{\alpha_{ic}+\beta_{ic}}=\frac{\alpha_{ic}^{0}+R_{ic}}{\alpha_{ic}^{0}+\beta_{ic}^{0}+R_{c}}\;.$$ (3)

Depending on how large the prior parameters $\alpha_{ic}^{0}$ and $\beta_{ic}^{0}$ are, this may be only a little different from the empirical read fraction estimate of *R*_*ic*_/*R*_*c*_, or it may be substantially different.

The variance of the posterior belief is one way of summarizing our uncertainty in that estimate, or in other words, a way of quantifying the precision of the measurement.

$$Var\left(p_{ic}|c\right)=\frac{\alpha_{ic}\beta_{ic}}{{\left(\alpha_{ic}+\beta_{ic}\right)}^{2}\left(\alpha_{ic}+\beta_{ic}+1\right)}=\frac{\left(\alpha_{ic}^{0}+R_{ic}\right)\left(\beta_{ic}^{0}+R_{c}-R_{ic}\right)}{{\left(\alpha_{ic}^{0}+\beta_{ic}^{0}+R_{c}\right)}^{2}\left(\alpha_{ic}^{0}+\beta_{ic}^{0}+R_{c}+1\right)}$$ (4)

Now, let us return to the question of correlating entity *i* with entity *j*. Instead of correlating *R*_*ic*_ with *R*_*jc*_ across conditions, or $p_{ic}^{emp}$ with $p_{jc}^{emp}$, we propose to correlate *p*_*ic*_ with *p*_*jc*_. The fact that we do not know *p*_*ic*_ or *p*_*jc*_ is no obstacle, as long as we can use our belief distribtions over them. In particular, we define the Bayesian correlation as

$$r_{ij}^{b}=\frac{Cov(p_{ic},p_{jc})}{\sqrt{Var\left(p_{ic}\right)Var\left(p_{jc}\right)}}$$ (5)

Here, the covariances and variances are taken with respect to both the condition *c* and the uncertainties represented in our posterior beliefs. Of course, if *i* = *j*, then the formula above reduces to *Var*(*p*_*ic*_)/*Var*(*p*_*ic*_) = 1. Otherwise, let us consider how to compute the different terms.

Using the Law of Total Covariance, we can decompose the covariance term into two parts, one which expresses the average covariance of *p*_*ic*_ and *p*_*jc*_ within conditions, and one which expresses the covariance of their posterior means across conditions.

$$Cov\left(p_{ic},p_{jc}\right)=E\left(Cov\left(p_{ic},p_{jc}|c\right)\right)+Cov\left(E\left(p_{ic}|c\right),E\left(p_{jc}|c\right)\right)$$ (6)

Here, we treat the condition *c* as a random variable, with each of its *k* possible values occuring with probability 1/*k*. The first expectation above is with respect to *c*, and the covariance term on the right is across conditions *c*. Above, we have described our beliefs over *p*_*ic*_ and *p*_*jc*_ as being given by independent Beta distributions. Thus, *Cov*(*p*_*ic*_, *p*_*jc*_|*c*) is zero by assumption. If we had chosen a Dirichlet distribution, however, then the covariance would be nonzero, and that term would have to be computed. To address the second term, let us introduce the overall mean true read fraction, averaged across conditions and over our belief for each condition.

$$E\left(p_{ic}\right)=\sum_{c=1}^{k}\frac{1}{k}E\left(p_{ic}|c\right)$$ (7)

Then, the second term in the covariance formula is given by the following.

$$Cov\left(E\left(p_{ic}|c\right),E\left(p_{jc}|c\right)\right)=\sum_{c=1}^{k}\frac{1}{k}\left(E\left(p_{ic}|c\right)-E\left(p_{ic}\right)\right)\left(E\left(p_{jc}|c\right)-E\left(p_{jc}\right)\right)$$ (8)

That resolves the numerator of the Bayesian correlation formula in Eq 5. For the denominator of Eq 5, we need to compute the variance of *p*_*ic*_. Similar to the covariance, the variance can be obtained by first applying the Law of Total Variance, dividing the variance into variability across conditions and variability (uncertainty) within each condition, as quanitified by the posterior.

$$Var\left(p_{ic}\right)=E\left(Var\left(p_{ic}|c\right)\right)+Var\left(E\left(p_{ic}|c\right)\right)$$ (9)

$$=\sum_{c=1}^{k}\frac{1}{k}Var\left(p_{ic}|c\right)+\sum_{c=1}^{k}{\left(E\left(p_{ic}|c\right)-E\left(p_{ic}\right)\right)}^{2}$$ (10)

The first term represents our uncertainty in the value of *p*_*ic*_ (see Eq 4) averaged across conditions *c*, while the second term represents variability in the mean of our belief across conditions. This resolves the denominator of the Bayesian correlation equation, completing our derivation of the formulas.

Below, we demonstrate Bayesian correlation on some real data. Just to concretize the discussion at this point, however, let us see how the Bayesian correlation plays out on the examples described in the Introduction. In our first example, we had gene expression counts *R*_*x*⋅_ = (1000, 1000, 10000), *R*_*y*⋅_ = (100, 100, 200) and *R*_*z*⋅_ = (0, 0, 1), with *R*_*c*_ = 10^6^ total sequencing depth in each condition. And as mentioned above, the Pearson correlation between any pair of genes in *r*^*p*^ = 1. This is true whether considering the raw read counts *R*_*ij*_ or the empirical read fractions $p_{ij}^{emp}$. However, the Bayesian correlations between these genes are $r_{xy}^{b}=0.971$, $r_{xz}^{b}=0.378$ and $r_{yz}^{b}=0.367$. Appropriately, the *xy* correlation is deemed highest, although not quite one, because of the small uncertainties in the *x* and *y* measurements. Also appropriately, the correlations of *x* and *y* to *z* are much smaller, because of the much greater uncertainty in the true levels of *z*. These calculations were done with uniform priors for the true read fractions, $\alpha_{ij}^{0}=\beta_{ij}^{0}=1$. In our second example, we had gene expression counts *R*_*w*⋅_ = *R*_*x*⋅_ = (10, 0, 0) and *R*_*y*⋅_ = *R*_*z*⋅_ = (0, 100, 0) out of total read counts *R*_*c*_ = (10^6^, 10^7^, 10^6^). In this example, the standard Pearson correlations of *w* with *x*, and of *y* with *z*, are one. However, the Bayesian correlations are $r_{wx}^{b}=0.859$ and $r_{0.948}^{b}$. It is sensible that posterior estimate of the *wx* correlation be lower than *yz* correlation, because the latter genes are measured with greater fidelity, being expression under a condition with greater read depth. For these simple examples at least, the Bayesian correlation produces a sensible reduction in estimated correlation towards zero, when there is more uncertainty in the true measured levels.

### Good priors for Bayesian correlation analysis

One of the most important steps in a Bayesian statistical analysis is the selection of a good prior—that is, a prior that contains good information of the process so the amount of data necessary to approximate well the exact solution is small, and a prior that does not overly bias the final result. In this section we consider some of the possible choices and we study which ones are best for our problem.

The simplest choice is $\alpha_{ic}^{0}=1$ and $\beta_{ic}^{0}=1$, for all entitites *i* and conditions *c*. With these parameters, the Beta distribution takes the form of a flat function in the interval [0, 1] (see Fig 1B). We denote by $r_{ij}^{b1}$ the Bayesian correlation obtained with this prior. The motivation for this choice is quite simple. A given read in an HTS dataset either is attributable to the entity *i* or not. Assuming we know nothing about entity *i* or the condition *c* before hand, a noninformative prior seems a plausible choice. However, this choice turns out to be problematic, especially for low count entities. To demonstrate this, let us turn to a real dataset.

#### Bayesian correlations based on the uniform prior can be highly biased

We decided to analyze the “Wang” dataset from the ReCount resource of analysis-ready RNA-seq gene count datasets [21, 22]. The ReCount resource conveniently summarizes expression data from a number of other datasets in terms of read counts attributed to genes. The Wang dataset contains gene expression data from 15 diverse human tissue samples (with some tissues represented by more than one sample). From this matrix, we eliminated genes with zero counts under every condition, and we eliminated identical rows, resulting in 10574 unique, expressed genes.

**Fig 2:**
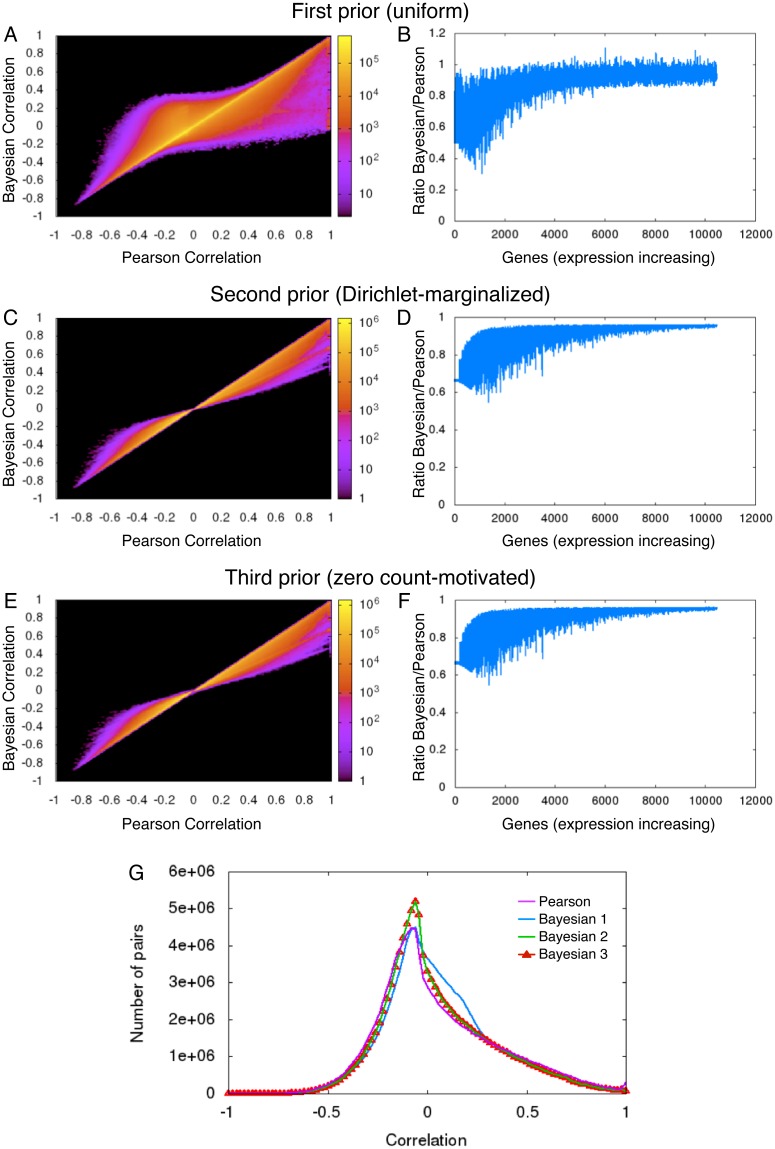
Analysis of different priors for Bayesian correlation analysis, on the reduced Wang dataset. (A) Scatter plot comparing traditional Pearson correlations with Bayesian correlations using a uniform prior for expression levels. (B) Average absolute correlation coefficient for different genes, with increasing expression along the x-axis. (C-D) Similar to panels A-B, but with the Dirichlet-inspired prior. (E-F) Similar to A-B, but with the zero count-inspired prior. (G) Histogram of all pairwise correlation coefficients (self-correlations omitted), for Pearson and all three Bayesian correlation analyses.

Fig 2A shows a scatter plot of the Bayesian and Pearson correlations for all 55,899,451 pairs of expressed genes. The density of points is indicated on a scale from low density (purple) to high density (yellow). There are many points on or near the *y* = *x* line, indicating pairs of genes for which the Bayesian and Pearson correlations agree. However, there is also a substantial number of points that are far from the *y* = *x* line. There are many pairs of genes that Pearson estimates as being highly correlated, at or near *r*^*p*^ = 1, whereas the Bayesian correlation is subtantial lower, even approximately zero. Inspection of some individual points reveals that these are pairs of genes where at least one has very low read counts that just happen to match up well with some high count gene—usually a gene expressed predominantly in a single tissue type. However, the converse is also true. There are a number of gene pairs where the Pearson correlation is at or near zero, and yet the Bayesian correlation is nonzero (although only as large about 0.2 to 0.3). There are even gene pairs where the Pearson correlation is negative (say around −0.3) and the Bayesian correlation is positive (say around +0.3).

Fig 2B shows for each gene *i*, sorted in order of increasing total read count, the ratio of the mean absolute Bayesian correlation with all the other genes to the mean absolute Pearson correlation with all other genes:

$$\frac{\frac{1}{m-1}\sum_{j\neq i}\left|r_{ij}^{b1}\right|}{\frac{1}{m-1}\sum_{j\neq i}\left|r_{ij}^{p}\right|}\;.$$ (11)

We see that the lower expression genes have smaller Bayesian correlations than Pearson correlations, on average. As expression grows larger, there is an increasing tending for Bayesian and Pearson correlations to be of the same magnitude. We would like to think of Bayesian correlations as similar to Pearson, but moderated towards zero when signal levels are uncertain. Yet, the plots show that this is not the whole story. As mentioned above, the two can be of opposite sign, and the Bayesian correlations can be of even greater magnitude than the Pearson correlation. What is the source of this phenomenon?

Consider, as a special case, an entity with strictly zero counts in every condition: *R*_*ic*_ = 0 for all *c*. As stated above, we have removed such genes from consideration. But, the analysis of their Bayesian correlations is particularly clear. With a uniform prior, the posterior mean estimate of *p*_*ic*_ = $\alpha_{ic}^{0}/\left(\alpha_{ic}^{0}+\beta_{ic}^{0}+R_{c}\right)$ = 1/(2+*R*_*c*_) ≈ 1/*R*_*c*_. Thus, despite the lack of any evidence regarding entity *i*, the posterior mean estimate can go up or down depending on the sequencing depth *R*_*c*_ of the experiment. When two different entities, *i* and *j* ≠ *i*, both have zero counts, their posterior means will be correlated. Indeed, in Eqs 18 and 19, the Bayesian correlation with this prior can be closely approximated by

$$r_{ij}^{b1}\approx \frac{\frac{1}{k}\sum_{c=1}^{k}{\left(\frac{1}{R_{c}}-\sum_{c^{\prime }=1}^{k}\frac{1}{k}\frac{1}{R_{c^{\prime }}}\right)}^{2}}{\left(\sum_{c=1}^{k}\frac{1}{k}\frac{1}{R_{c}^{2}}\right)+\sum_{c=1}^{k}\frac{1}{k}{\left(\frac{1}{R_{c}}-\sum_{c^{\prime }=1}^{k}\frac{1}{k}\frac{1}{R_{c^{\prime }}}\right)}^{2}}\;.$$ (12)

We can see that if some *R*_*c*_’s are very different from the mean, the numerator of Eq (12) is large and, therefore, the correlation is not zero.

#### Two priors that minimize spurious bias between low count entities

In order to avoid the problem presented by the uniform prior, we present two possible alternatives. Clearly the process of attributing reads to entities is not one of flipping a coin. In any practical problem the number of entities *m* (e.g., genes) is bigger than two. Thus, for any read, the prior probability that it comes from gene *i* should be $\frac{1}{m}$, not $\frac{1}{2}$. One way to capture this is with a Beta distribution with prior parameter values $\alpha_{ic}^{0}=\frac{1}{m}$ and $\beta_{ic}^{0}=1-\frac{1}{m}$. This choice could also be justified as the marginalization of a Dirichlet distribution $D(\alpha_{1}=\frac{1}{m},$ $\alpha_{2}=\frac{1}{m},$ …, $\alpha_{m}=\frac{1}{m})$. Any prior with parameters proportional to these would also produce the same prior mean, but we make this choice because it offers a weak prior that does not overly influence the posterior estimates. We call this our second choice of prior, and denote by $r_{ij}^{b2}$ the Bayesian correlation obtained with this prior.

Finally, we present a third prior specifically designed to avoid the problem of spurious correlations between zero-count entities (and by similarity, between low-count entities): $\alpha_{ic}^{0}=\frac{R_{ic}+1}{R_{max}+1},\beta_{ic}^{0}=1$, where $R_{max}=\underset{i}{\text{max}}\;R_{i}$. We denote by $r_{ij}^{b3}$ the Bayesian correlation obtained with this prior. As we reiterate formally below, the interesting property of this prior is that if *R*_*ic*_ = 0 for all *c*, or *R*_*jc*_ = 0 for all *c*, then $r_{ij}^{b3}$ is exactly zero (see Theorem below and proof in Appendix A). Although this prior requires some information related with the data, namely the total read counts in each condition, it does not rely on the entities’ individual read counts, and thus can be considered a valid prior.

In Fig 2C–2E, we compare Bayesian correlations computed with our second and third priors to the Pearson correlations, just as we did for the first prior. We see that the second and the third priors perform better than the first prior, in the sense that they appear moderated towards zero compared to the Pearson correlations. This is especially true when the read counts are low. Fig 2G shows histograms of all the correlation coefficients for Pearson and Bayesian analyses with the three different choices of priors. We see that there are more high (> 0.7) Pearson correlations than there are Bayesian correlations with any choice of prior. We also see that the bulk of the correlations, which hover around −0.1 for Pearson correlation, are closer to zero for Bayesian correlation with second or third choices of prior. In contrast, Bayesian correlations with the first prior have a concentration just below zero and another concentration around 0.25–0.3.

**Fig 3:**
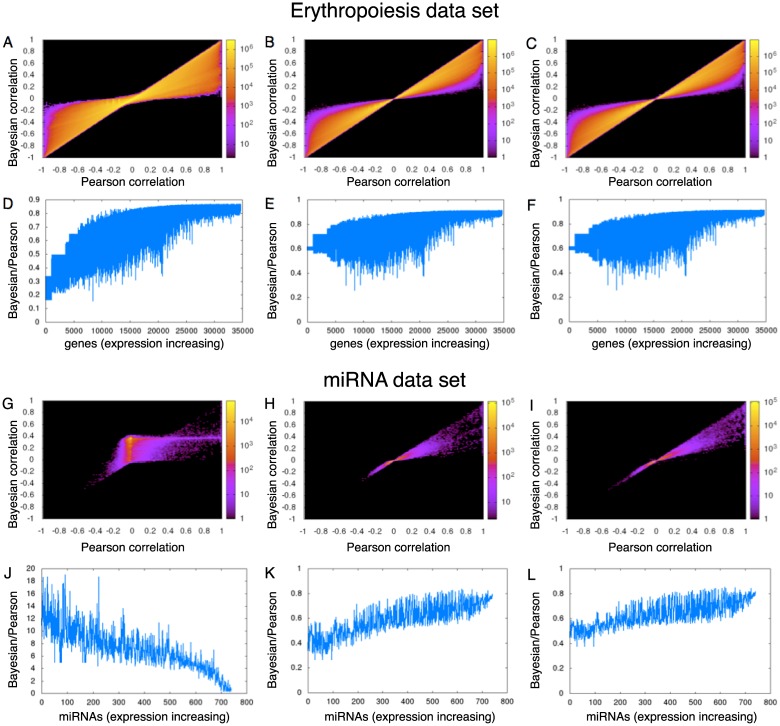
Comparison of Bayesian versus Pearson correlation analysis for first (left), second (middle) and third Bayesian priors (right) in erythropoeisis and miRNA datasets. (A-C) Bayesian versus Pearson correlations for erythropoiesis, using first, second and third priors respectively. (D-F) Ratios of mean Bayesian to Pearson correlations for erythropoiesis as a function of increasing gene expression. (G-I) Bayesian versus Pearson correlations for the miRNA dataset. (J-L) Ratios of mean Bayesian to Pearson correlations.

While the behavior of Bayesian correlation with second or third priors on the Wang dataset is satisfying, it is important to know whether there is any generality to the results. To further evaluate performance, we tested the method on two additional datasets. One is an RNA-seq gene expression time-series take at four days (4, 8, 11 and 14) spanning an erythropoiesis differentiation protocol, with each day sampled in duplicate [23]. We treat the replicates as individual conditions, although an alternative would have been to pool the reads from replicates. A comparison of the Bayesian and Pearson correlations can be found in Fig 3A–3F. As in the previous dataset, we see that Pearson and Bayesian correlations often agree, but there are also many pairs of genes for which the Bayesian correlation is moderated towards zero compared to the Pearson correlation. This is true for all three choices of priors. In contrast with the previous dataset, we do not see the first, uniform prior generating spurious non-zero correlations when the Pearson correlation is zero.

The third dataset we tried is a miRNA-seq study of the expression of 1143 precursor and mature microRNAs in 172 normal and cancerous human tissues, available in Table S5 of Landgraf *et al.* [24]. The results of Bayesian and Pearson correlation analyses can be seen in Fig 3G–3L. While the second and third Bayesian priors perform as expected, there is a major problem with spurious positive correlations when using the first Bayesian prior (Fig 3G). This is due to the vast differences in sequencing depth between different samples. In this early study, the average sequencing depth per condition was just 912 reads per condition, and ranged from 60 to 2162. As a result, when looking at the ratio of mean absolute Bayesian correlations to mean absolute Pearson correlations (Fig 3J), we see that for low expression miRNAs the Bayesian correlation is much higher than the Pearson correlation, on average. However, Bayesian analysis with the second or third priors does not suffer this problem.

In order to establish the generality of these observations, we analyzed Bayesian correlations with different priors theoretically. The following Theorem, proved in Appendix A, shows that the second and third priors have properties of correctly suppressing correlations between low-count entities for any dataset.

**Theorem 1** *Let R be the count matrix for m entities under k conditions. Let i and j ≠ i be two entities. Let n*_*i*_ = max_*c*_ *R*_*ic*_, *and similarly for n*_*j*_. *Let k*_1_, *k*_2_, *k*_3_ *and k*_4_ *be, respectively, the number of conditions for which: R*_*ic*_ = 0 *and R*_*jc*_ > 0; *R*_*ic*_ > 0 *and R*_*jc*_ = 0; *R*_*ic*_ = 0 *and R*_*jc*_ = 0; *and R*_*ic*_ > 0 *and R*_*jc*_ > 0. *Then, if n*_*i*_ = 0 *and n*_*j*_ = 0, *we have*

1. $r_{ij}^{b3}=0$, *and*
2. $r_{ij}^{b2}\approx \frac{1}{m}r_{ij}^{b1}\;.$

*If* *n*_*i*_ > 0 *or* *n*_*j*_ > 0, *then*

- $|r_{ij}^{b3}|\leq n_{i}n_{j}\frac{4R_{max}^{2}}{kR_{min}^{2}}(\frac{k_{1}}{k}+\frac{k_{2}}{k}+\frac{k_{3}}{k^{2}}+k_{4}).$

Note that $r_{ij}^{b1}\in \left[-1,1\right]$ and typically *m* ≫ 1. Therefore, priors 2 and 3 both suppress the artificial correlations from strictly zero-count entities. And, for low-count entities (i.e., when *n*_*i*_ and *n*_*j*_ are small compared to the number of conditions, hence read counts are zero under most conditions) then Bayesian correlation with the third prior is provably small.

### Kernel property and use in clustering

In machine learning, a kernel is any way of measuring similarity between objects that can be expressed as an inner product in some (possibly infinite dimensional) feature space [25, 26]. It is an important property because there are many machine learning algorithms that are designed to work with any kernel, including kernel-based clustering, kernel principal components analysis, classification by support vector machines, and so on. Regardless of choice of priors, it turns out that the Bayesian correlation, like the Pearson correlation, is a kernel. The proof is relatively straightforward and not of independent interest, but can be found in Appendix B.

**Theorem 2** *Given any count matrix R and entity- and condition-specific priors α*_*ic*_, *β*_*ic*_ > 0, *the Bayesian correlation as defined above consititutes a kernel*.

Although this is true for any choice of priors, in this section we focus on the third notion of prior defined above, and compare the behaviour of Pearson and Bayesian correlations in clustering the Wang data.

#### Agreement between Bayesian and Pearson correlations

**Fig 4:**
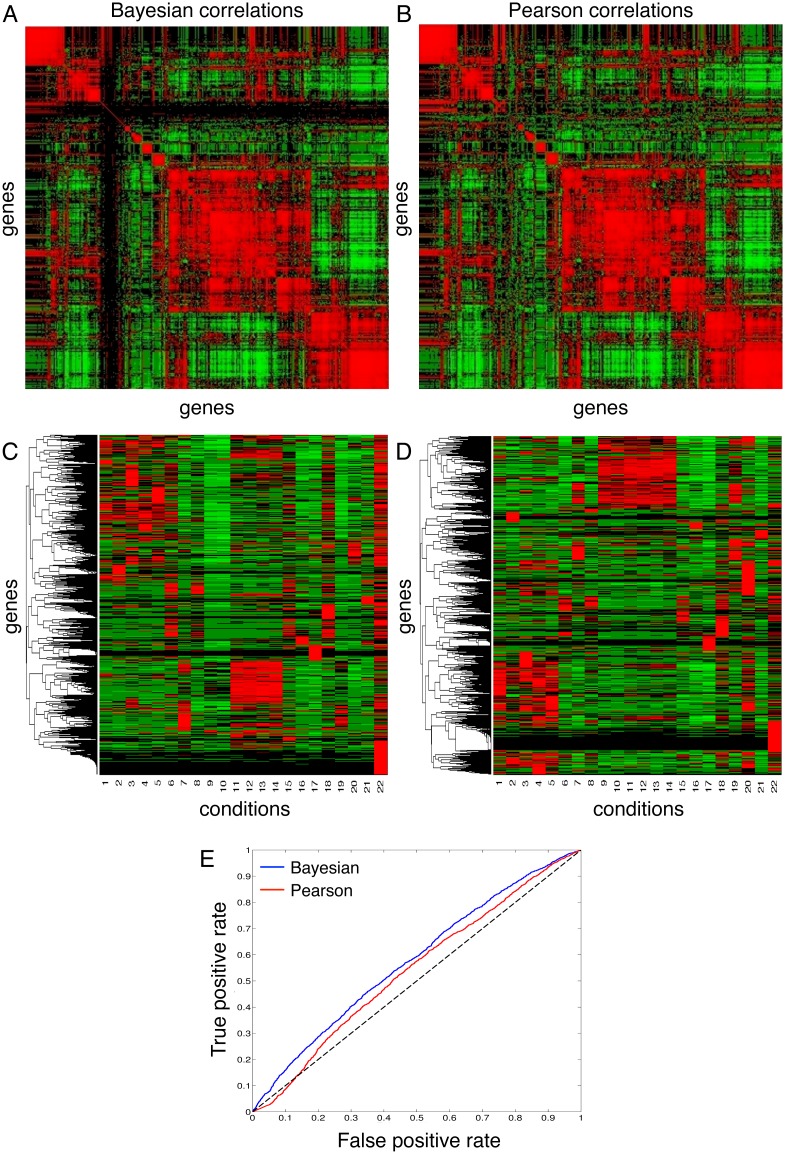
Clustering using Bayesian correlation as a similarity measure. (A-B) Heatmaps for Bayesian (prior 3) and Pearson correlations between genes in the Wang dataset, using the same row- and column-orderings for both. (C-D) Hierarchical clustering of genes by one minus the Bayesian or Pearson correlation respectively. (E) ROC curve for genes with tissue-specific GO terms, ordered by decreasing maximum correlation to any other gene.

To further assess how the Bayesian and Pearson correlation schemes compare, we plotted the correlation coefficients between the genes in the Wang dataset (omitting genes with zero counts) and arranged them into square matrices. For easy visualization, we then applied hierarchical row and column clustering to the Bayesian matrix using the Euclidean distance metric and average linkage. The Pearson matrix was then reordered to reflect the same ordering scheme of the clustered Bayesian matrix. The rearranged matrices are shown in Fig 4A and 4B. Clearly, both the Bayesian and the Pearson correlations schemes agree to a great extent. They show essentially the same block structures along the main diagonal, and similar relationships away from the main diagonal. The most prominent difference in the plots is that the Bayesian heatmap has a cluster of genes with mutual correlations near zero, producing near-black vertical and horizontal bars. The corresponding part of the Pearson plot shows a mixture of positive and negative correlations. These are low expression genes for which the Bayesian scheme discounts apparent correlations due to uncertainty in true expression levels.

#### Hierarchical clustering using the correlations as distance metrics

A very useful application of the kernel property of the Bayesian correlation function is its use as a similarity measure for clustering. As a demonstration, we carried out hierarchical clustering of the rows of the Wang data matrix (resulting in clustering genes) using one minus the pairwise Bayesian correlation as the distance metric. (Our software takes distance rather than similarity matrices. The third prior was employed). For comparison, in parallel, we also clustered the genes using one minus the Pearson correlation as the distance metric. Fig 4C and 4D display the clustergram heatmaps for the two cases, where the colors map to standardized expression levels (read counts). There are many similarities between the two clusterings. Both include a set of genes enriched in samples 1 to 5, and another group enriched in samples 11 to 14. Sample 22 is notable for high expression of genes that are unexpressed or weakly expressed in other samples.

To further assess the biological relevance of the results of the Bayesian and the Pearson correlation methods, we used the Wang dataset and the corresponding correlation coefficients as scores to predict the role of a gene in a tissue-specific biological process. We downloaded the Biological Process (BP) Complete Gene Ontology (GO) terms for all the genes in the dataset from the PANTHER database (http://pantherdb.org/citePanther.jsp). Out of a total of 10574 genes (after pre-processing the Wang dataset as described earlier), PANTHER found matches for 10506 genes, thus ignoring 68 genes from the list. We reasoned that genes with strong tissue-specific expression patterns would likely be similar to at least some other tissue-specific genes. Thus, a Bayesian and a Pearson correlation score was assigned to each gene by finding the highest value at which the gene correlates with any other gene in the dataset. Using this score as a predictor and a moving threshold value, we built receiver operatic characteristic (ROC) curves for the two correlation metrics. The ground truths were taken to be the genes that have at least one of the following tissue-specific word stems corresponding to the tissues in the Wang dataset: “adipose”, “brain”, “breast”, “neur”, “cereb”, “heart”, “liver”, “lymph”, “musc”, “testes”, “hepat”, “cardi”, “arter”, “mamm”, “sperm”, “epidid”, “intes”. (We verified that each stem matched only appropriate, tissue-specific GO terms). From the ROC curves, it is clear that the Bayesian correlation metric serves as a better predictor for tissue-specific co-expression of genes, or in other words, participation of a given gene in a tissue-specific biological process. Specifically, it has superior performance on the highest score genes, where it has eliminated low-expression genes to which the Pearson correlation falls pray.

## Discussion

In this paper, we have developed a Bayesian approach to correlation analysis of entities measured by high-throughput sequencing. Its central feature is that the precision of measurements are taken into account. In particular, Bayesian estimates of true signal levels allow us to quantify uncertainty due to both differing true signal levels between entities and due to different sequencing depths between experiments. In empirical results on a multi-tissue RNA-seq gene expression dataset, we found that the Bayesian correlation approach acts as intended. For low-expression genes, whose true expression levels are less well measured in the signal-to-noise-ratio sense, correlations are naturally suppressed in a graded manner, without resorting to some arbitrary expression-level or significance cut off. Conversely, for high-expression genes, for which measurements are relatively certain, Bayesian and Pearson correlation estimates closely agree. Although we have demonstrated the approach so far only on an RNA-seq dataset, the same approach could be used for many other types of data. miRNA-seq, ChIP-seq and Methyl-seq data, for example, could also be analyzed by the same approach, as well as correlated with each other.

Beyond empirical results, we have also established several key theoretical properties of our Bayesian correlation estimates. First, to our initial surprise, we found that assuming a uniform prior over signal levels can result in highly biased correlation estimates, especially for low-count entities. By understanding the source of this problem, however, we were able to propose two alternative priors, both of which are provably well behaved, in the sense of producing zero or near-zero correlations for low-count entities. We also proved that, regardless of the choice of prior, the Bayesian correlation constitutes a kernel, and thus a valid similarity measure that can be used in any number of kernel-based machine learning algorithms. We put this to use by showing a Bayesian correlation-based hierarchical clustering of the RNA-seq data, and comparing it to Pearson-based clustering.

Despite our positive results, there is more work to be done in the area of Bayesian correlation analysis for sequencing data. For one thing, we assume only a set of independent conditions, and a desire to compute correlations between entities across those conditions. We have not considered the issue of replicate experiments, besides simply treating them as additional conditions. Treated this way, the number of replicates seems to have little effect on either Bayesian or Pearson correlations (see simulation study in Appendix D). However, one may want to “average out” variability within replicates, and compute correlations only across groups of replicates. Relatedly, we have only modeled uncertainty resulting from a generic model of randomly sampling reads from a pool in which the entities are fractionally represented. However, in RNA-seq analysis especially, and ChIP-seq analysis to some degree, there are more sophisticated noise models in use, attempting to account for biological variability, PCR-amplification artifacts, and so on. Thus, a more comprehensive treatment of Bayesian correlation analysis would allow for a more general class of random sampling models.

Another major generalization of the current work would be to consider other measures of similarity. For instance, Spearman correlation and mutual information [27, 28] are two other often-used methods of measuring the similarity of gene expression profiles. These measures can differ significantly from Pearson correlation (see Appendix E for Spearman correlation applied to the datasets in this paper). Yet, like Pearson correlation, they can yield artificially high similarities under certain conditions. A fundamental contribution of the present paper is to suggest incorporating measurement uncertainty into the similarity metric, and we showed how to do this in the case of Pearson correlation. Figuring out how to do this for other similarity measures is an important direction for future work.

## Appendix A: Proof of Theorem 1

Our empirical results show that priors 2 and 3 suppress artificial correlations. In this appendix we give some bounds on the Bayesian correlations. The proofs are straightforward, but tedious. For the sake of convenience, we introduce the following notation.

$$\begin{array}{lll} E_{1} & = & \{c\text{ such that }R_{ic}=0\text{ and }R_{jc}\neq 0\},\\ E_{2} & = & \{c\text{ such that }R_{jc}=0\text{ and }R_{ic}\neq 0\},\\ E_{3} & = & \{c\text{ such that }R_{ic}=0\text{ and }R_{jc}=0\},\\ E_{4} & = & \{c\text{ such that }R_{ic}\neq 0\text{ and }R_{jc}\neq 0\},\\ f_{ic} & = & \frac{\alpha_{ic}^{0}+R_{ic}}{\alpha_{ic}^{0}+\beta_{ic}^{0}+R_{c}},\\ l_{ic} & = & \frac{R_{ic}}{\alpha_{ic}^{0}+\beta_{ic}^{0}+R_{c}},\\ f_{i} & = & \sum_{c=1}^{k}\frac{1}{k}\frac{\alpha_{ic}^{0}+R_{ic}}{\alpha_{ic}^{0}+\beta_{ic}^{0}+R_{c}},\\ f_{i}^{j} & = & \sum_{c\in E_{j}}\frac{1}{k}\frac{\alpha_{ic}^{0}+R_{ic}}{\alpha_{ic}^{0}+\beta_{ic}^{0}+R_{c}},\\ l_{i}^{j} & = & \sum_{c\in E_{j}}\frac{1}{k}\frac{R_{ic}}{\alpha_{ic}^{0}+\beta_{ic}^{0}+R_{c}},\\ k_{j} & = & |E_{j}|. \end{array}$$

With the new notation, the formula for the covariance reads

$$Cov\left(p_{ic},p_{jc}\right)=\frac{1}{k}\sum_{l}\sum_{c\in E_{l}}\left(f_{ic}-f_{i}^{1}-f_{i}^{2}-f_{i}^{3}-f_{i}^{4}\right)\left(f_{jc}-f_{j}^{1}-f_{j}^{2}-f_{j}^{3}-f_{j}^{4}\right).$$ (13)

It is possible to obtain a simplified expression for the covariance with the third prior. The original formula for the covariance is

$$\begin{array}{cc} Cov\left(p_{ic},p_{jc}\right)=\frac{1}{k}\sum_{c=1}^{k} & \left(\frac{\alpha_{ic}^{0}+R_{ic}}{\alpha_{ic}^{0}+\beta_{ic}^{0}+R_{c}}-\sum_{c^{\prime }=1}^{k}\frac{1}{k}\frac{\alpha_{ic^{\prime }}^{0}+R_{ic^{\prime }}}{\alpha_{ic^{\prime }}^{0}+\beta_{ic^{\prime }}^{0}+R_{c^{\prime }}}\right)\\ & \left(\frac{\alpha_{jc}^{0}+R_{jc}}{\alpha_{jc}^{0}+\beta_{jc}^{0}+R_{c}}-\sum_{c^{\prime }=1}^{k}\frac{1}{k}\frac{\alpha_{jc^{\prime }}^{0}+R_{jc^{\prime }}}{\alpha_{jc^{\prime }}^{0}+\beta_{jc^{\prime }}^{0}+R_{c^{\prime }}}\right). \end{array}$$

Note that some terms can be spliced.

$$\begin{array}{c} \left(\frac{\alpha_{ic}^{0}+R_{ic}}{\alpha_{ic}^{0}+\beta_{ic}^{0}+R_{c}}-\sum_{c^{\prime }=1}^{k}\frac{1}{k}\frac{\alpha_{ic^{\prime }}^{0}+R_{ic^{\prime }}}{\alpha_{ic^{\prime }}^{0}+\beta_{ic^{\prime }}^{0}+R_{c^{\prime }}}\right)=\\ \frac{\alpha_{ic}^{0}}{\alpha_{ic}^{0}+\beta_{ic}^{0}+R_{c}}-\sum_{c^{\prime }=1}^{k}\frac{1}{k}\frac{\alpha_{ic^{\prime }}^{0}}{\alpha_{ic^{\prime }}^{0}+\beta_{ic^{\prime }}^{0}+R_{c^{\prime }}}+\\ \frac{R_{ic}}{\alpha_{ic}^{0}+\beta_{ic}^{0}+R_{c}}-\sum_{c^{\prime }=1}^{k}\frac{1}{k}\frac{R_{ic^{\prime }}}{\alpha_{ic^{\prime }}^{0}+\beta_{ic^{\prime }}^{0}+R_{c^{\prime }}}. \end{array}$$ (14)

With the third prior, the first two terms are cancelled. Recall

$$\begin{array}{ccc} \alpha_{ic}^{0} & = & \frac{R_{c}+1}{R_{max}+1},\\ \beta_{ic}^{0} & = & 1. \end{array}$$

Thus,

$$\frac{\alpha_{ic}^{0}}{\alpha_{ic}^{0}+\beta_{ic}^{0}+R_{c}}=\frac{\frac{R_{c}+1}{R_{max}+1}}{\frac{R_{c}+1}{R_{max}+1}+1+R_{c}}=\frac{\left(R_{c}+1\right)\frac{1}{R_{max}+1}}{\left(R_{c}+1\right)\frac{1}{R_{max}+1}+1}=\frac{\frac{1}{R_{max}+1}}{\frac{1}{R_{max}+1}+1}=\frac{1}{2+R_{max}}.$$

Therefore,

$$\begin{array}{cc} & \frac{\alpha_{ic}^{0}}{\alpha_{ic}^{0}+\beta_{ic}^{0}+R_{c}}-\sum_{c^{\prime }=1}^{k}\frac{1}{k}\frac{\alpha_{ic^{\prime }}^{0}}{\alpha_{ic^{\prime }}^{0}+\beta_{ic^{\prime }}^{0}+R_{c^{\prime }}}\\ = & \frac{1}{2+R_{max}}-\sum_{c^{\prime }=1}^{k}\frac{1}{k}\frac{1}{2+R_{max}}=0. \end{array}$$ (15)

Therefore, for the third prior the formula for the covariance is

$$Cov^{3}\left(p_{ic},p_{jc}\right)=\frac{1}{k}\sum_{m}\sum_{c\in E_{m}}\left(l_{ic}-l_{i}^{1}-l_{i}^{2}-l_{i}^{3}-l_{i}^{4}\right)\left(l_{jc}-l_{j}^{1}-l_{j}^{2}-l_{j}^{3}-l_{j}^{4}\right).$$ (16)

### Analysis in the case of zero counts

In this section, we analyse the case in which the total number of read counts is zero for a row. According to Eq (25), it is trivial that if all *R*_*ic*_ = 0, then the covariance of the third prior is zero. Therefore,

$$r_{ij}^{b3}\left(p_{ic},p_{jc}\right)=0\;,$$ (17)

and we have proved the first property of the Theorem stated in the main manuscript.

The covariance of the first prior is

$$\begin{array}{cc} Cov^{1}\left(p_{ic},p_{jc}\right) & =\sum_{c=1}^{k}\frac{1}{k}{\left(\frac{1}{1+1+R_{c}}-\sum_{c^{\prime }=1}^{k}\frac{1}{k}\frac{1}{1+1+R_{c^{\prime }}}\right)}^{2}\\ & \approx \sum_{c=1}^{k}\frac{1}{k}{\left(\frac{1}{R_{c}}-\sum_{c^{\prime }=1}^{k}\frac{1}{k}\frac{1}{R_{c^{\prime }}}\right)}^{2}, \end{array}$$ (18)

and its variance is given by

$$\begin{array}{ccc} Var^{1}\left(p_{ic}\right) & = & \left(\sum_{c=1}^{k}\frac{1}{k}\frac{1(1+R_{c})}{{\left(1+1+R_{c}\right)}^{2}\left(1+1+R_{c}+1\right)}\right)+\\ & & \sum_{c=1}^{k}\frac{1}{k}{\left(\frac{1}{1+1+R_{c}}-\sum_{c^{\prime }=1}^{k}\frac{1}{k}\frac{1}{1+1+R_{c^{\prime }}}\right)}^{2}\\ & \approx & \left(\sum_{c=1}^{k}\frac{1}{k}\frac{1}{R_{c}^{2}}\right)+\sum_{c=1}^{k}\frac{1}{k}{\left(\frac{1}{R_{c}}-\sum_{c^{\prime }=1}^{k}\frac{1}{k}\frac{1}{R_{c^{\prime }}}\right)}^{2}. \end{array}$$ (19)

The covariance of the second prior is

$$\begin{array}{ccc} Cov^{2}\left(p_{ic},p_{jc}\right) & = & \sum_{c=1}^{k}\frac{1}{k}{\left(\frac{\frac{1}{m}}{\frac{1}{m}+\frac{m-1}{m}+R_{c}}-\sum_{c^{\prime }=1}^{k}\frac{1}{k}\frac{\frac{1}{m}}{\frac{1}{m}+\frac{m-1}{m}+R_{c^{\prime }}}\right)}^{2}\\ & = & \sum_{c=1}^{k}\frac{1}{k}{\left(\frac{\frac{1}{m}}{1+R_{c}}-\sum_{c^{\prime }=1}^{k}\frac{1}{k}\frac{\frac{1}{m}}{1+R_{c^{\prime }}}\right)}^{2}\\ & = & \frac{1}{m^{2}}\sum_{c=1}^{k}\frac{1}{k}{\left(\frac{1}{1+R_{c}}-\sum_{c^{\prime }=1}^{k}\frac{1}{k}\frac{1}{1+R_{c^{\prime }}}\right)}^{2}\\ & \approx & \frac{1}{m^{2}}\sum_{c=1}^{k}\frac{1}{k}{\left(\frac{1}{R_{c}}-\sum_{c^{\prime }=1}^{k}\frac{1}{k}\frac{1}{R_{c^{\prime }}}\right)}^{2}\\ & = & \frac{1}{m^{2}}Cov^{1}\left(p_{ic},p_{jc}\right). \end{array}$$ (20)

and the variance is

$$\begin{array}{ccc} Var^{2}\left(p_{ic}\right) & = & \left(\sum_{c=1}^{k}\frac{1}{k}\frac{\left(\frac{1}{m}\right)\left(\frac{m-1}{m}+R_{c}\right)}{{\left(\frac{1}{m}+\frac{m-1}{m}+R_{c}\right)}^{2}\left(\frac{1}{m}+\frac{m-1}{m}+R_{c}+1\right)}\right)+\\ & & \sum_{c=1}^{k}\frac{1}{k}{\left(\frac{\frac{1}{m}}{\frac{1}{m}+\frac{m-1}{m}+R_{c}}-\sum_{c^{\prime }=1}^{k}\frac{1}{k}\frac{\frac{1}{m}}{\frac{1}{m}+\frac{m-1}{m}+R_{c^{\prime }}}\right)}^{2}\\ & = & \sum_{c=1}^{k}\frac{1}{k}\frac{\left(\frac{1}{m}\right)\left(\frac{m-1}{m}+R_{c}\right)}{{\left(1+R_{c}\right)}^{2}\left(1+R_{c}+1\right)}+\\ & & \sum_{c=1}^{k}\frac{1}{k}{\left(\frac{\frac{1}{m}}{1+R_{c}}-\sum_{c^{\prime }=1}^{k}\frac{1}{k}\frac{\frac{1}{m}}{1+R_{c^{\prime }}}\right)}^{2}\\ & = & \frac{1}{m}\sum_{c=1}^{k}\frac{1}{k}\frac{1(\frac{m-1}{m}+R_{c})}{{\left(1+R_{c}\right)}^{2}\left(1+R_{c}+1\right)}+\\ & & \sum_{c=1}^{k}\frac{1}{k}\frac{1}{m}{\left(\frac{1}{1+R_{c}}-\sum_{c^{\prime }=1}^{k}\frac{1}{k}\frac{1}{1+R_{c^{\prime }}}\right)}^{2}\\ & \approx & \frac{1}{m}\sum_{c=1}^{k}\frac{1}{k}\frac{1}{R_{c}^{2}}+\sum_{c=1}^{k}\frac{1}{k}{\left(\frac{1}{R_{c}}-\sum_{c^{\prime }=1}^{k}\frac{1}{k}\frac{1}{R_{c^{\prime }}}\right)}^{2}\\ & \approx & \frac{1}{m}Var^{1}\left(p_{ic}\right). \end{array}$$ (21)

Therefore,

$$r_{ij}^{b2}\left(p_{ic},p_{jc}\right)=O\left(\frac{1}{m}\right)\;r_{ij}^{b1}\left(p_{ic},p_{jc}\right).$$ (22)

This concludes the proof of the second property.

### Analysis in the case of non-zero read counts

In this section we consider a more general case. Given two different entities *i* and *j*, let

$$n_{i}=\max_{c}R_{ic},$$ (23)

$$n_{j}=\max_{c}R_{jc}.$$ (24)

The covariance for the third prior is given by

$$Cov^{3}\left(p_{ic},p_{jc}\right)=\frac{1}{k}\sum_{m}\sum_{c\in E_{m}}\left(l_{ic}-l_{i}^{1}-l_{i}^{2}-l_{i}^{3}-l_{i}^{4}\right)\left(l_{jc}-l_{j}^{1}-l_{j}^{2}-l_{j}^{3}-l_{j}^{4}\right).$$ (25)

Note that some of that terms are zero, so that

$$\begin{array}{cc} Cov^{3}\left(p_{ic},p_{jc}\right) & =\;\frac{1}{k}\sum_{c\in E_{1}}\left(-l_{i}^{2}-l_{i}^{4}\right)\left(l_{jc}-l_{j}^{1}-l_{j}^{4}\right)+\frac{1}{k}\sum_{c\in E_{2}}\left(l_{ic}-l_{i}^{2}-l_{i}^{4}\right)\left(-l_{j}^{1}-l_{j}^{4}\right)+\\ & \;\frac{1}{k}\sum_{c\in E_{3}}\left(-l_{i}^{2}-l_{i}^{4}\right)\left(-l_{j}^{1}-l_{j}^{4}\right)+\frac{1}{k}\sum_{c\in E_{4}}\left(l_{ic}-l_{i}^{2}-l_{i}^{4}\right)\left(l_{jc}-l_{j}^{1}-l_{j}^{4}\right). \end{array}$$

Note that

$$|\left(-l_{i}^{2}-l_{i}^{4}\right)\left(l_{jc}-l_{j}^{1}-l_{j}^{4}\right)\left|\leq \right|\left(-l_{i}^{2}-l_{i}^{4}\right)\max\left\{l_{jc},-l_{j}^{1}-l_{j}^{4}\right\}|\leq \left(n_{i}+n_{i}\right)\left(n_{j}+n_{j}\right).$$ (26)

Thus,

$$\begin{array}{cc} |Cov^{3}\left(p_{ic},p_{jc}\right)| & \leq \;\frac{1}{k}\left(\frac{k_{1}}{k}\frac{2n_{i}}{R_{min}}\frac{2n_{j}}{R_{min}}+\frac{k_{2}}{k}\frac{2n_{i}}{R_{min}}\frac{2n_{j}}{R_{min}}+\frac{k_{3}}{k^{2}}\frac{2n_{i}}{R_{min}}\frac{2n_{j}}{R_{min}}+k_{4}\frac{2n_{i}}{R_{min}}\frac{2n_{j}}{R_{min}}\right)\\ & =\;\frac{4n_{i}n_{j}}{kR_{min}^{2}}\left(\frac{k_{1}}{k}+\frac{k_{2}}{k}+\frac{k_{3}}{k^{2}}+k_{4}\right). \end{array}$$ (27)

The last step is to obtain a lower bound for the variance.

$$\begin{array}{cc} Var^{3}\left(p_{ic}\right) & =\;\left(\sum_{c=1}^{k}\frac{1}{k}\frac{\left(\alpha_{ic}^{0}+R_{ic}\right)\left(\beta_{ic}^{0}+R_{c}-R_{ic}\right)}{{\left(\alpha_{ic}^{0}+\beta_{ic}^{0}+R_{c}\right)}^{2}\left(\alpha_{ic}^{0}+\beta_{ic}^{0}+R_{c}+1\right)}\right)+\\ & \;\sum_{c=1}^{k}\frac{1}{k}{\left(\frac{\alpha_{ic}^{0}+R_{ic}}{\alpha_{ic}^{0}+\beta_{ic}^{0}+R_{c}}-\sum_{c^{\prime }=1}^{k}\frac{1}{k}\frac{\alpha_{ic^{\prime }}^{0}+R_{ic^{\prime }}}{\alpha_{ic^{\prime }}^{0}+\beta_{ic^{\prime }}^{0}+R_{c^{\prime }}}\right)}^{2} \end{array}$$ (28)

$$=\;D\left(p_{ic}\right)+Cov\left(p_{ic},p_{ic}\right)\;,$$ (29)

where

$$\begin{array}{cc} D\left(p_{ic}\right) & =\;\sum_{c=1}^{k}\frac{1}{k}\frac{\alpha_{ic}^{0}\left(\beta_{ic}^{0}+R_{c}-R_{ic}\right)}{{\left(\alpha_{ic}^{0}+\beta_{ic}^{0}+R_{c}\right)}^{2}\left(\alpha_{ic}^{0}+\beta_{ic}^{0}+R_{c}+1\right)}\\ & \;+\sum_{c=1}^{k}\frac{1}{k}\frac{R_{ic}\left(\beta_{ic}^{0}+R_{c}-R_{ic}\right)}{{\left(\alpha_{ic}^{0}+\beta_{ic}^{0}+R_{c}\right)}^{2}\left(\alpha_{ic}^{0}+\beta_{ic}^{0}+R_{c}+1\right)}\\ & \approx \;\sum_{c=1}^{k}\frac{1}{k}\frac{\alpha_{ic}^{0}R_{c}}{R_{c}^{2}R_{c}}+\sum_{c=1}^{k}\frac{1}{k}\frac{R_{ic}R_{c}}{R_{c}^{2}R_{c}}\\ & \geq \;\frac{1}{k}\sum_{c=1}^{k}\frac{R_{c}^{2}}{R_{max}R_{c}^{3}}+\frac{k_{2}+k_{4}}{k}\frac{R_{c}}{R_{c}^{3}}\\ & \geq \;\frac{1}{R_{max}^{2}}. \end{array}$$ (30)

In this way, we obtain that the Bayesian correlation is bounded by

$$|r_{ij}^{b3}\left(p_{ic},p_{jc}\right)|\leq n_{i}n_{j}\frac{4R_{max}^{2}}{kR_{min}^{2}}\left(\frac{k_{1}}{k}+\frac{k_{2}}{k}+\frac{k_{3}}{k^{2}}+k_{4}\right).$$

## Appendix B: Proof of the kernel property

Our proof of the kernel property is divided in two steps. The first step concerns the correlation of two different entities (*i* ≠ *j*). Define

$$\begin{array}{ccc} g_{ic} & = & E\left(p_{ic}|c\right)-E\left(p_{ic}\right)\\ g_{i} & = & \left(g_{i1},g_{i2},\ldots ,g_{ik}\right)\\ V_{i} & = & Var\left(p_{ic}\right)\\ S_{i} & = & \sqrt{V_{i}}\\ \phi_{i} & = & g_{i}/S_{i} \end{array}$$

In words, *g*_*ic*_ is the difference between our posterior mean estimate of the true read fraction for entity *i* in condition *c*, *p*_*ic*_, and the average of those estimates across conditions. This term occurs in Eq 6 where, multiplied with a corresponding term for entity *j*, it contributes to the covariance computation. *g*_*i*_ is the vector of those terms across all *k* conditions. *V*_*i*_ is the variance of *p*_*ic*_, with respect to our beliefs and across conditions, or in other words, the quantity given by Eq 10. Thus, *ϕ*_*i*_ is the vector of deviations of our condition-specific mean estimates of *p*_*ic*_ from the overall mean estimate, normalized by the standard deviation of those estimates. With this notation, the reader may verify that the Bayesian correlation in Eq 5 can be written as

$$r_{ij}^{b}=<\phi_{i},\phi_{j}>\;,\;\;\;\;$$ (31)

where < ⋅, ⋅ > denotes the inner product. Therefore, for different entities *i* ≠ *j*, the Bayesian correlation can be written as an inner product in a suitable feature space.

To proceed further, we consider the case *i* = *j*, which requires a slight modification. The reason for this is that the formula above only works when the first term on the right hand side of Eq 8 is zero. This is true by assumption when *i* ≠ *j*, but it is most definitely not true when *i* = *j*. Indeed, when *i* = *j* that first term is such that the numerator and denominator of Eq 5 are equal, and thus the self-correlation is one. In contrast, if one blindly applied the formula above to compute the correlation of an entity with itself, one would generally get an answer less than one.

To acount for this, we consider each entity to be represented not only by its number of counts under the different conditions, but also by a unique identifying label in *L* = {1, 2, …, *M*}, where *M* is the number of entities. Then, for entity *i* in $R^{k}\times L$ we propose the feature mapping:

$$\begin{array}{ccc} \psi_{i} & : & \left({\mathbb{R}}^{k}\times L\right)\to {\mathbb{R}}^{k}\times {\mathbb{R}}^{M}\\ & = & \left(\phi \left(p_{i}\right),0,\ldots ,0,\underset{{\left(i+1\right)}^{st}\;\text{element}}{\underset{︸}{\sqrt{1-<\phi \left(p_{i}\right),\phi \left(p_{i}\right)>}}},0,\ldots ,0\;\right). \end{array}$$

If *i* ≠ *j*, then <*ψ*_*i*_, *ψ*_*j*_ > = < *ϕ*_*i*_, *ϕ*_*j*_>. However, if *i* = *j*, then <*ψ*_*i*_, *ψ*_*j*_> = <*ψ*_*i*_, *ψ*_*i*_> = $<\phi_{i},\phi_{i}>-{\left(\sqrt{1-<\phi \left(p_{i}\right),\phi \left(p_{i}\right)>}\right)}^{2}$ = 1. So, in either case, we can express the Bayesian correlation of two entities *i* and *j* (where *j* may or may not be the same as *i*) as an inner product in a certain feature space. This concludes the proof that the Bayesian correlation is a kernel.

## Appendix C: Computational complexity and run-time

Pearson and Bayesian correlation computations have the same order of computational complexity. Recall that we have *m* entities and *k* conditions, and we start with an *m* × *k* matrix of read counts *R*. The equations in the main text describe how to compute the Pearson and Bayesian correlations between a single pair of entities, *A* and *B*. However, in the experiments in this paper, and often in practice, we want to compute all pairwise correlations. We will analyze the complexity of both computations.

For the Pearson correlation, we first need to compute the empirical read fractions, or equivalently, divide each column of *R* by its own column sum. This takes *O*(*mk*) operations. Even if we are only interested in correlating one pair of entities, the complexity is the same, because of the need of computing the row sums. Then, for each entity, we need to compute its mean read fraction across conditions, taking *O*(*k*) per entity or *O*(*mk*) for all. Similarly, we need to compute the variance of the empirical read fractions across conditions, taking *O*(*k*) per entity or *O*(*mk*) for all. For a pair of entities, computing the covariance across conditions takes *O*(*k*) time, thus *O*(*m*^2^ *k*) for all pairs. Computing the correlation based on the covariance(s) and the variances takes *O*(1) time per pair, thus *O*(*m*^2^) for all pairs. All together, the complexity for correlating one pair of entities is *O*(*mk*) and the complexity for correlating all pairs of entities is *O*(*m*^2^ *k*).

For Bayesian correlation, the story is similar. We need to know the total read counts per condition, which takes *O*(*mk*) to obtain, so that we can compute the posterior *α* and *β* values—whether we are interested in a pair of entities or all entities. Then, computing posterior means and variances takes *O*(*k*) time per entity, or *O*(*mk*) for all entities. The covariance of the posterior means takes *O*(*k*) per entity pair, and thus *O*(*m*^2^ *k*) for all pairs. Finally, the correlation takes *O*(1) per entity pair or *O*(*m*^2^) for all. Thus, identical to the case for Pearson correlation, we require *O*(*mk*) to correlate a single pair or *O*(*m*^2^ *k*) to correlate them all.

In our R-language implementation, the computations can be represented in terms of vectors and matrices, which allows software- and/or hardware-level parallelism to speed the computations compared to a naive loop-based implementation. For the Bayesian computation, for example, we begin by producing *m* × *k* matrices of the priors *α*^0^ and *β*^0^, along with a matrix *R*^*c*.*s*.^ where each element in column *c* equals the sum of column *c* in the read count matrix *R*. From these, the posteriors are obtained as *α* = *α*^0^ + *R* and *β* = *β*^0^ + *R*^*c*.*s*.^ − *R*. The posterior mean matrix is *M* = *α*/(*α*+*β*), where the division is element-wise. The cross-conditional posterior means are given by the row sums of *M*, which we then place into an *m* × *k* matrix *M*^*r*.*s*.^ with *k* identical values on each row. Letting *Z* = *M* − *M*^*r*.*s*.^, the covariances are obtained as *C* = *MM*^*T*^, where the superscript *T* denotes the matrix transpose. The variances can be obtain similarly. As an example, computing all pairwise Bayesian correlations for the Wang dataset of over 10,000 genes using our R code (see S1 Code) on a Macbook Pro with 2.6 GHz Intel Core i7 processor and 16 GB RAM takes approximately 3.1 seconds.

## Appendix D: Simulation study on the influence of numbers of replicates

As mentioned in the main text, our approach to Bayesian correlation analysis does not account for replicate data, except to treat replicates as additional conditions. As a partial test of how the number of replicates influences both Pearson and Bayesian correlations, we simulated additional replicates for the Erythropoiesis dataset. Recall that the original data comprises two replicates of four biological conditions, which we treat as eight conditions. From each of those eight conditions, we resampled, with replacement, the same number of reads four different times. We then analyzed: a 16-condition dataset comprising the original eight conditions and resampled versions of the same, a 24-condition dataset comprising the original eight plus two resampled versions, a 32-condition dataset comprising the original eight plus three resampled versions, and a 40-condition dataset comprising the original eight plus four resampled versions.

**Fig 5:**
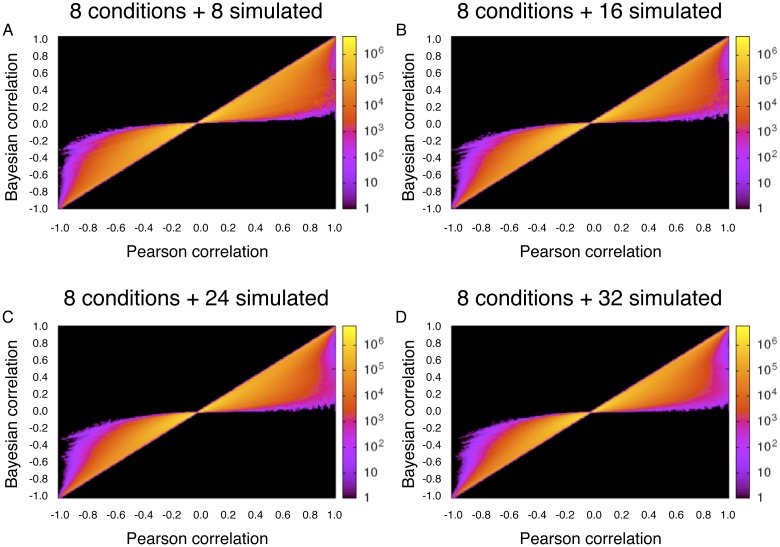
Bayesian versus Pearson correlations with increasing numbers of simulated replicates of the Erythropoiesis data, both of which seem largely insensitive to the number of replicates.

We computed Pearson and Bayesian correlations, using the third prior, for each of these augmented datasets. The results are shown in Fig 5. Visually, the additional, simulated replicates have little effect on Bayesian or Pearson correlations. As a more formal analysis, we first assessed the similarity of the Pearson and Bayesian correlations, in the original eight conditions, across all 602,131,753 pairs of genes. To assess similarity, we computed the Pearson correlation coefficient between the Pearson and Bayesian correlations for all gene pairs. In the original eight conditions, this turned out to be 0.9540. With eight, 16, 24 and 32 additional simulated replicates, the similarity between Pearson and Bayesian correlations increased to 0.9643, 0.9692, 0.9719 and 0.9738 respectively. Thus, with these simulated replicates, and on this specific dataset, the effect is only to increase modestly the agreement between Pearson and Bayesian correlation estimates. There remain many gene pairs where the Bayesian correlation is substantially moderated towards zero compared to the Pearson correlation estimate.

## Appendix E: Comparison to Spearman correlation

**Fig 6:**
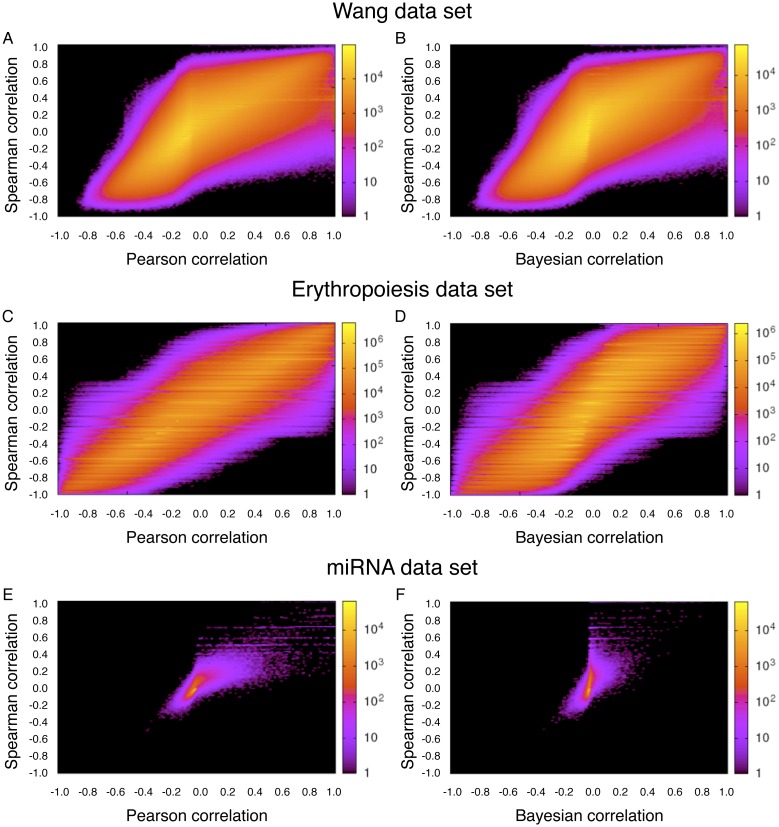
Spearman correlations compared to Pearson and Bayesian (third prior) correlations, on each of the three datasets.

We computed Spearman (rank) correlations for the three datasets studied above. Fig 6 shows the results, in comparison with Pearson correlations and Bayesian correlations computed using the third prior. The Spearman correlations are quite different from either Bayesian or Pearson correlations. Compare also to Figs 2E, 3C and 3I.

## Supporting Information

S1 Code R code implementing Bayesian correlation computations. (R)
